# Association of pro-fibrinolytic receptor AnnexinA2 with tissue plasminogen activator/Inhibitor-1 in pre-eclampsia

**DOI:** 10.4314/ahs.v23i1.33

**Published:** 2023-03

**Authors:** Komal Ruikar, Vitthal Khode, Shilpa S Shetty, E Sarathkumar, Prakash Patil, Satish Patil, Anil Bargale, Roshni sadashiv, Praveenkumar Shetty

**Affiliations:** 1 Department of Physiology, SDM College of Medical Sciences & Hospital, Shri Dharmasthala Manjunatheshwara University, Dharwad, India; 2 Department of Biochemistry/Central Research Lab, K S Hegde Medical Academy, Nitte (Deemed to be University) Mangaluru, India; 3 Department of Biochemistry, SDM College of Medical Sciences & Hospital, Shri Dharmasthala Manjunatheshwara University, Dharwad, India; 4 Department of Anatomy, SDM College of Medical Sciences & Hospital, Shri Dharmasthala Manjunatheshwara University, Dharwad, India

**Keywords:** Pre-eclampsia, fibrinolysis, AnxA2, tPA, PAI-1

## Abstract

**Background:**

The clinical manifestations of pre-eclampsia are related to placental anti-angiogenic factor alteration. These variations are mainly due to the alteration of plasminolytic components. The study aims to compare the expression of plasminolytic components in the placenta of women with and without pre-eclampsia.

**Material and Methods:**

The study included pregnant women with pre-eclampsia as PE group (n = 30) and without pre-eclampsia as a control group (n = 30). Placental bed biopsy tissues were collected. AnxA2, tPA, PAI-1 expression in the placental villous tissue was quantitatively evaluated using immunohistochemistry, western blot, and real time-PCR analysis.

**Results:**

The results of the study showed a significant decrease in the expression of ANXA2 and increased expression of tPA and PAI-1 in PE group compared to control group (p<0.005). AnxA2 expression showed positive correlation with tPA (r=+0.895, p=0.002) and negative correlation with PAI-1(r=-0.905, p=0.020) in control group whereas in the PE group AnxA2 expression was negatively correlated with tPA ((r=-0.801, p=0.016) and PAI-1 (R=-0.831, P=0.010).

**Conclusion:**

Decreased AnxA2 with increased expression of PAI-1 and tPA may be responsible for the altered fibrinolytic activity and play a significant role in pre-eclampsia pathogenesis.

## Introduction

Pre-eclampsia (PE) is a multifactorial pregnancy-specific disorder characterized by hypertension and proteinuria after 20 weeks of pregnancy^1^. Pre-eclampsia affects about 2–8% of all pregnancies and is the principal cause of maternal and fetal morbidity and mortality worldwide^2^. Although PE's exact etiology is still undetermined, some evidence shows that failure of trophoblast invasion of maternal uterine spiral arteries subsequently reduces placental blood flow and is the actual cause of this complication. A large body of literature has confirmed placental and fetal hypoxia's effect on endothelial cell dysfunction and pre-eclampsia pathogenesis^3^. The most established pathophysiology in pre-eclampsia resides in the uterus' abnormal trophoblastic implantation with reduced placental perfusion. The secondary pathology seems to be endothelial cell injury, oxidative stress, and the coagulation system^4^. Placental dysfunction plays a major role in pre-eclampsia pathogenesis as well as in modulating the disease outcome. The deposition of fibrin and microthrombi is the major histo-pathogenic abnormality in the placental dysfunction of pre-eclampsia^5^. The mechanism of these thrombotic changes in the placenta are not fully understood. Most studies have focused on the clotting mechanisms, and very little is known about the changes in the fibrinolytic mechanism in pregnancies of complicated pre-eclampsia^6^,^7^.

In the fibrinolytic mechanism, tPA delivers a fundamental role in regulating fibrin's breakdown containing thrombi by converting clot-bound plasminogen to active plasmin enzymatically which in turn is involved in fibrinogenolysis^8^. Recent studies have revealed that tPA interacts with cellular receptors like AnxA2 that allow it to carry out additional biological functions and activate specific signal transduction pathways^9^. The cell surface fibrinolysis is the concept of fibrinolytic association, in which the tPA-dependent conversion of plasminogen to active plasmin is precisely organized through the formation of a multimolecular compound consisting of tPA, the AnxA2, and plasminogen^10^. AnxA2 is a cell-surface protein, which, in complex with its binding partner p11, forms the receptor for both tPA, the inactive precursor of plasmin, and its activator, plasminogen. By assembling tPA, AnxA2, and plasminogen, this complex increases the tPA's catalytic efficiency, at least 60 times more efficiently than the same amount of tPA alone^11^. PAI-1 is the key inhibitor of fibrinolysis compared to PAI-2 and PAI-3 during pregnancy^12^, accounting for 60% of the PA-inhibitory activity in the plasma^13^. With this study background, we hypothesize that as Anx2-tPA-PAI-1 factors are associated with the placental bed's fibrinolytic system, their expression may be altered in pregnancies complicated with pre-eclampsia when compared to normal pregnancies. Therefore, the present study aims to compare the expression of plasminolytic components (AnxA2, tPA, and PAI-1) in the placental bed of pregnant women with and without pre-eclampsia.

## Material and Methods

Current study is an observational case-control study. After obtaining institutional ethical clearance, 60 pregnant women undergoing cesarean delivery were recruited for the study with informed consent and further divided into two groups. The control group had pregnant women without pre-eclampsia (n=30; normotensive), and the PE group included pregnant women with pre-eclampsia (n=30). A detailed case history was taken. Body Mass Index (BMI) was calculated. Pre-eclampsia was diagnosed based on increased blood pressure (140/90 mmHg) in a pregnant woman after 20 weeks of amenorrhea, accompanied by proteinuria (0.3 g/24 h or 1+dipstick reading), as per the guidelines of the American College of Obstetricians and Gynecologists^14^.

PE can have an early onset starting before 34 weeks of gestation and late onset after 34 weeks of gestational age and can be classified as mild or severe, depending on the severity of the symptoms present^15^.

Mild PE is characterized by hypertension with systolic blood pressure ≥140 mmHg and diastolic blood pressure ≥90 mm Hg accompanied with proteinuria ≥0.3 g per 24 h after 20 weeks of gestational age in a previously normotensive parturient. Severe PE is characterised by a systolic blood pressure ≥160 mmHg or and diastolic blood pressure ≥110 mmHg, and proteinuria >5 g per 24 h along with disturbances in central nervous system, epigastric pain, liver dysfunction and fetal growth restriction. The PE women group was divided, according to the aforementioned criteria, into two groups; mild PE group (15 patients) and severe PE (15 patients).

### Inclusion criteria

Placenta of normotensive and different severity of preeclampsia of a women having age group of 19 to 35 were included.

### Exclusion criteria

Patients with chronic hypertension, gestational diabetes, renal disease, collagen vascular disease, epilepsy and other pregnancy complications like fetal anomalies or chromosomal abnormalities were excluded from the study.

Fresh placental bed biopsy tissues were obtained from 60 term pregnancies at the time of cesarean delivery. The expression of AnxA2, tPA and PAI-1 was analysed using immunohistochemistry, western blot, and real-time PCR.

### Immunohistochemistry Analysis

Villous parenchyma (1 cm3 each) from the central and marginal regions of part of the placental disc were collected in 4-5 biopsies using a scalpel. 3µm thick sections were obtained from formalin-fixed and paraffin-embedded placental tissues. The levels of AnxA2, tPA and PAI-1 proteins in the 60 placental villous tissues were analysed by immunohistochemistry as per the method described previously^15^. In brief, the sections were heated in 0.01 M citrate buffer solution (pH 6.0) in a water bath at 98° C for 20 min, followed by deparaffinization and endogenous peroxidase blockage. Then incubated with the mouse monoclonal antibody to AnxA2 (BD Biosciences) tPA and PAI-1 (Santa Cruz Biotechnology) at 1:100 dilution at 4° C overnight, and visualized with 3,3′-diaminobenzidine (DAB) detection kit (Vector Labs). Anti-rabbit and anti-mouse IgG whole molecule (Sigma-Aldrich) was used at a dilution of 1:1000 for negative control. Two pathologists analysed IHC-stained samples, and all samples were blinded. The staining intensity of proteins graded at a 0 (no staining) scale to 3+ (strong stain). The positivity of AnxA2, tPA and PAI-1 was scored based on the percentage of positive cells: 0 = 0 percent of positively stained cells; 1, weakly stained tissue or 1–25 percent of positive cells; score 2 = moderate stained tissue, or 26–50 percent of positively stained cells; and score 3, strongly stained tissue or more than 50% of stained cells^16^.

### Western blot analysis

Total protein was extracted and quantitated for the expression analysis in the placental tissue, as described previously^13^. Using Tris-HCl buffer, the total protein (40µg) was separated using 10% Bis-Tris PAGE gel. The antibodies were used against AnxA2 (1: 1000, monoclonal mouse, BD Biosciences, Cat. No. 9061), tPA (1: 1000, Santa Cruz Biotechnology, SC-7269), PAI-1 (1: 1000, Santa Cruz Biotechnology, SC-5297), and GAPDH (1: 1000, Santa Cruz Biotechnology, SC-166574). Appropriate secondary antibodies conjugated to horseradish peroxidase (Bio-Rad) were incubated with respective membranes for 2 hours at room temperature. The membranes were developed using ECL plus (Bio-Rad), and the image was captured using an enhanced Chemi-luminescence system, G: BOX Chemi XX6/XX9. Immunoblot for GAPDH was considered as the internal loading control. The protein bands were quantified and normalized relatively as the control band with Image J, version 1.35d (National Institutes of Health Image software).

### RNA preparation, RT-PCR, and real-time PCR

Total RNA was extracted using TRIzol reagent (Invitrogen, Carlsbad, CA, USA). Complementary DNA (cDNA) was synthesized from 2µg of total RNA, using the Takara cDNA synthesis kit. Template cDNA was subjected to PCR amplification using gene-specific sense and antisense primers (Table No. 1). RT-PCR conditions were at 95°C for 5 min, followed by 40 cycles of 95°C for 30 s, annealing at 60°C for the 30s, and extension at 72°C for 30 s in a thermal cycle (Quant Studio 5 by Applied Biosystems). The quantitative amount of each gene was standardized against the house-keeping gene β-actin. The RNA levels were expressed as a ratio, using the ‘delta-delta’ method to compare the relative expression results between normotensive control and PE groups 17.

**Table 1:** Sequence of primers used for reverse transcriptasepolymerase chain reaction (RT-PCR)

Gene	Sequence
**AnxA2**	5′-CTGGCAAAGGGTAGAAGAGCA-3′ 5′-CGTCATAGAGATCCCGAGCAT-3′
**tPA**	5′-AGGAGCCAGATCTTACCAAGTGA-3′ 5′-CGCAGCCATGACTGATGTTG-3′
**PAI-1**	5′-GGCCATTACTACGACATCCTG-3′ 5′-GGTCATGTTGCCTTTCCAGT-3′
**β-actin**	5′-GGGAAATCGTGCGTGACATTAAG-3′ 5′-TGTGTTGGCGTACAGGTCTTTG-3′

### Statistical analysis

Statistical analysis was carried out by using Graph Pad Prism version 7.04. To compare parameters including clinic-demographic characteristics and expression profiles between the groups, students' t-test and Mann-Whitney U-test were used. Pearson's coefficient was used to correlate AnxA2, tPA and PAI-1 expression within the groups and also to check the association of AnxA2 tPA and PAI-1 with maternal and fetal parameter. A two-tailed P<0.05 was considered to indicate a statistically significant difference.

## Results

Age and BMI showed statistically insignificant differences between the PE and control groups. Compared with the normotensive control group, the systolic and diastolic blood pressures were significantly higher in patients with PE, and the baby's weight was reduced. There was a statistically significant decrease in mean gestational age in PE than the control group. The platelet count was reduced and prothrombin time was increased in pre-eclamptic group (P<0.05) as shown in Table 2.

**Table 2:** Clinico-demographic characteristics of study subjects examined

Clinical data	Preeclampsia (N=30)	Normotensive (N=30)	P-value
Maternal Age (years)	25.65±2.9	25.53±3.3	0.8913
BMI (kg/m2)	26.62±1.6	25.65±2.2	0.102
Gestational age (weeks)	35.73±0.29	37.67±0.18	0.0001*
Birth Weight (Kgs)	2.59±0.06	2.97±0.05	0.0001*
Placental weight (gms)	486±10.16	503±18.4	0.183
Gravidity	1±0.0	2.3±0.6	0.000*
Parity	1±0.0	1.3±0.4	0.007*
SBP (mm Hg)	157.8±8.8	106.6±6.7	0.000*
DBP (mm Hg)	94.33±4.3	71.1±6.8	0.000*
Platelet s (103/µL)	2.46±0.66	2.64±0.04	0.02*
Prothrombin time(seconds)	13.5±0.7	12.0±0.3	0.000*

* p<0.05 was considered statistically significant.

### Histopathological examination

**Figure 1:**
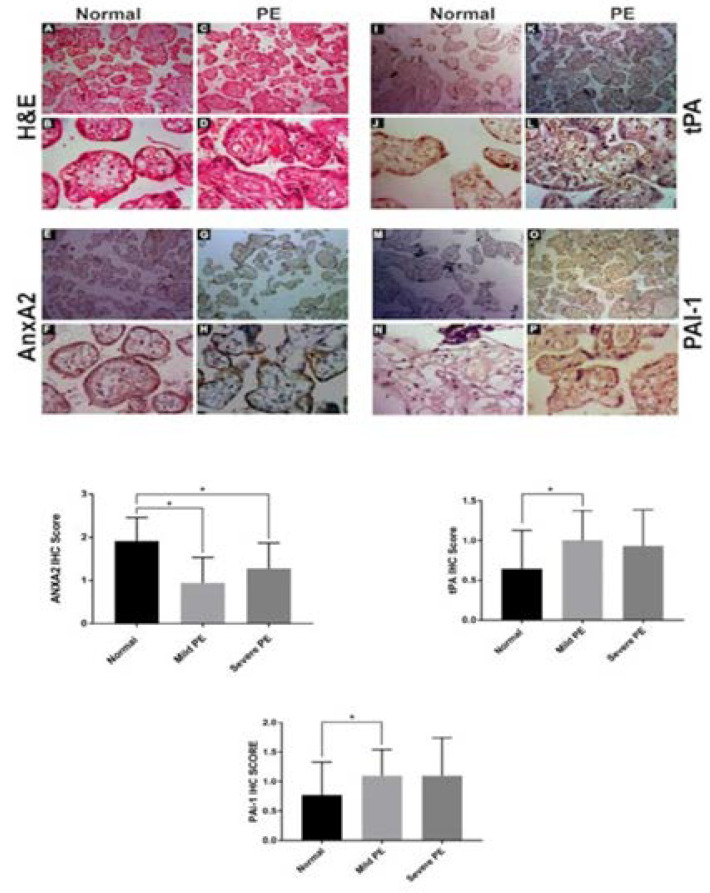
Representative microscopic image showing the histology of Control (A, B) and PE (C, D) placenta. Immunohistochemistry of placental villi with primary antibodies AnxA2 in normal (E, F) and PE placenta (G, H). (Note the more intense staining of AnxA2 in normal placenta compared to moderate staining in PE placenta). Representative immunohistochemistry of tPA in normal (IJ) and PE placenta (K, L). tPA is localized to the syncytiotrophoblast and up-regulated in the pre-eclamptic placenta. No staining was observed in the control group placenta (IJ). PAI-1 expression in normal (M, N) and PE (O, P) and up-regulated in PE placenta. Magnification 10X, bar =200 µm and 40X, bar =

Microscopic examination revealed that the number, volume, and density of placental villi in PE group were increased compared to the control group. In contrast, the surface area of the placental villous and villi diameter is reduced in PE group compared to control group. Other villous abnormality observed in PE placenta was increased stromal fibrosis, fibrinoid necrosis, thickening of cytotrophoblast basement membrane and increased syncytial knot formation as demonstrated in Figure 1.

### Immunoactivity of AnxA2, tPA and PAI-1 in placental villous tissue and their association with maternal and fetal parameter

Immunostaining of placental villous tissue showed reduced expression of AnxA2 in the PE group compared to the control group. Several different cell compartments in both placental bed biopsies of the PE and control groups were positive for AnxA2 including the membrane of syncytiotrophoblastic cells, chorionic villous stromal cells and endothelial cells. A statistically significant decrease in the expression was observed in the membrane of syncytiotrobhoblast, chorionic villous stromal cells and villous vascular endothelial cells in PE group (Figure 1. G, H) compared with placenta of normal pregnancies (Figure 1. E, F).

Representative image of IHC of tPA (Figure 1. I, J, K, L) and PAI-1 (Figure 1. M, N, O, P) is shown under low and high power. We have noticed the tPA and PAI- 1 expression is increased in PE placental villi compared to the normal placenta indicated with the black arrowhead.

Qualitative assessment of immunoreactivity revealed that the AnxA2 protein level was decreased in the placenta of PE compared to the placenta of normotensive women (P= 0.01). A statistically significant decrease in the expression was observed in membrane of syncytiotrobhoblast, chorionic villous stromal cells and villous vascular endothelial cells in PE group (Mild and Severe P=0.04, 0.01) in syncytiotrophoblast compared with placentas of normal pregnancies. The expression of AnxA2 the placentas of severe PE is slightly more than in mild PE however not statistically significant (P=0.993). But irrespective of severity, AnxA2 is decreased in PE group.

Most of the cases were negative for tPA in the normal placenta where weak expression was detected in syncytiotrophoblast membrane and villous stromal cells in pre-eclamptic placenta (P= 0.04). Qualitative analysis of PAI- 1 revealed weaker staining in the control group placenta than in the PE group (mild and severe PE) (P=0.01). Qualitative analysis of tPA and PAI- 1 revealed weaker staining in the placentas of the control group than that in the PE group (mild and severe PE) (P< 0.005). The correlation analysis between AnxA2 with maternal parameter prothrombin time revealed the presence of strong negative correlation (r=-0.301 P=0.004), whereas there was no significant correlation was observed with either tPA (r=-0.144, P=0.174) or PAI-1(r= -0.042 P=0.317). Additionally, AnxA2 expression showed the positive correlation with the birth weight of the baby (r=0.280 P= 0.019) and tPA is negatively correlated with birth weight of the baby (r=-0.255 P=0.033) while no correlation existed with PAI-1 expression.

### Expression of AnxA2, tPA and PAI-1 in placenta

**Figure 2:**
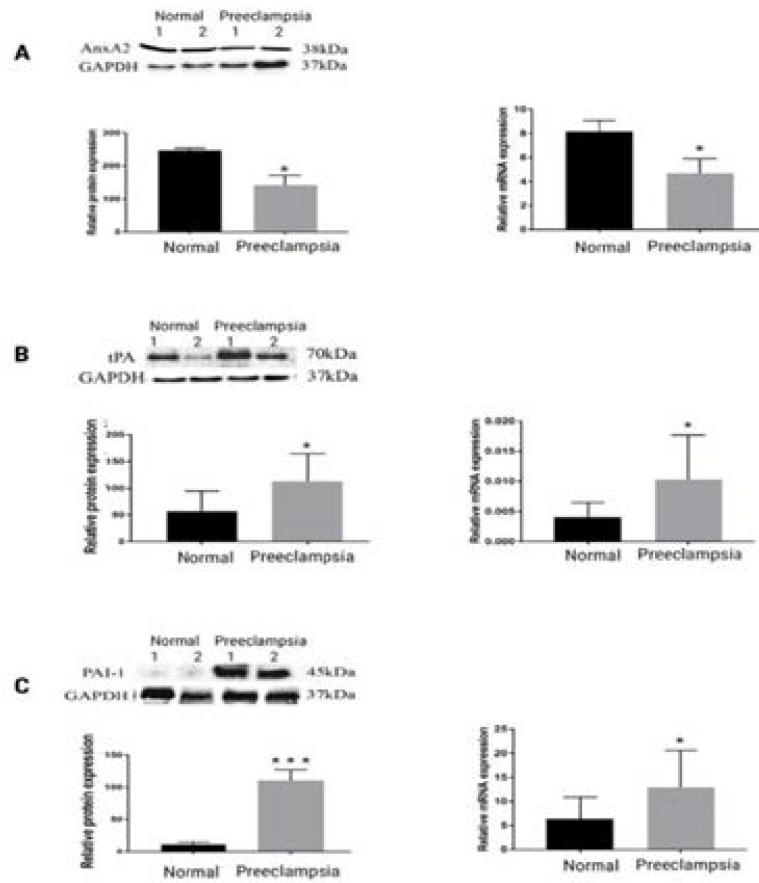
Western blot and RT-PCR analysis showed that AnxA2 in PE placenta were significantly reduced and tPA/PAI-1 expression was increased on protein and mRNA levels.

Western blotting was also performed because the technique of IHC does not lend itself to quantification. Expression levels of proteins were confirmed by densitometry. AnxA2 expression was significantly decreased in pre-eclamptic placentas by 1.88-fold in PE compared to normal placenta (P = 0.0001). tPA and PAI- 1 expression increased by 1.7-fold and 9.7-fold respectively (P= 0.045, 0.0001). Therefore, the increment of PAI-1 is much greater than the increment of tPA in PE as shown in Figure 2.

### Expression of AnxA2, tPA and PAI-1 mRNA in placenta

The expression profiles of AnxA2, tPA and PAI-1 were examined by RT-PCR analysis. AnxA2 expression decreased by 1.8-fold, tPA was increased in pre-eclamptic placenta compared to the normal placenta by 2.5-fold (P=0.0105), and PAI-1 is increased 2.03-fold (P=0.0182) in pre-eclamptic placenta compared to the normal placenta. Levels of mRNAs are expressed as arbitrary units. An unpaired t-test was used to evaluate the statistical difference.

### Relationship between AnxA2 with tPA and PAI-1 expression

AnxA2 expression was positively correlated with tPA (r=+0.895, p=0.002) and negatively correlated with PAI-1(r=-0.905, p=0.020) in controls. AnxA2 expression was negatively correlated with tPA ((r=-0.801, p=0.016) and PAI-1 (R=-0.831, P=0.010) in PE.

## Discussion

The placenta plays a crucial role in pre-eclampsia pathogenesis, particularly in the severe onset form of the syndrome. The initial insult occurs early at the placentation site with shallow endovascular trophoblastic invasion and defective remodelling of the maternal spiral arteries, which leads to placental insufficiency caused by dysfunctional perfusion 18. Placental dysfunction plays a vital role in the pathogenesis and prognosis of pre-eclampsia 19. The imbalance of hemostasis observed in normal pregnancy seems to be increased in pre-eclampsia^20^. Endothelial cell damage or activation is said to play an essential role in pre-eclampsia and may cause the hemostatic changes observed in this syndrome^21^. While normal endothelial cells participate in hemostasis regulation, perturbed vascular cells may express prothrombotic changes promoting pathologic events^22^.

In the current study, in the pre-eclamptic placenta, the expression of AnxA2 decreased while tPA and PAI-1expression increased. These expression results showed consistency in immunohistochemistry, western blotting, and RT-PCR analysis. A significant correlation existed between the expression of AnxA2, tPA and PAI-1 in the placenta. In the normal placenta, AnxA2 expression showed a positive correlation with tPA and negative correlation with PAI-1, whereas in the pre-eclamptic placenta, AnxA2 expression was negatively correlated with tPA and PAI-1. Annexin A2 is negatively correlated with prothrombin time. This may be because of the altered physiology in pre-eclampsia, in particular the changes related to fibrinolysis.

The fibrinolytic mechanism is a unique process rigidly controlled by a series of cofactors, inhibitors, and receptors of the plasminolytic components^23^. During normal pregnancy, the overall fibrinolytic mechanism is depressed. Fibrinolysis activators tPA and uPA gradually increase and which is balanced by increased levels of PAI-1, a key regulator of fibrinolysis in-vivo^24^. One of the key mediators involved in the conversion of plasminogen to plasmin is AnxA2. This pro-fibrinolytic molecule serves as a surface receptor protein that binds to plasminogen and its activator, tPA functioning as a cofactor for plasmin generation and localizing fibrinolytic activity to the cell surface^25^. Plasmin is a highly reactive enzyme that is physiologically involved in fibrinolysis and plays a vital role in neo-angiogenesis. In the present study, microscopic and immunohistochemical analyses showed diffused fibrin deposits in the placenta with pre-eclampsia than in non-pre-eclamptic placenta^26^.

In this study, IHC analysis showed that AnxA2 was majorly expressed on the syncytiotrophoblast cell membrane, stromal cells, and villous vascular endothelial cells in the placental villi normal pregnant women. We observed that the expression of AnxA2 protein was much lower in pregnant women placenta with pre-eclampsia than normal pregnancies. Subsequently, the name of AnxA2 mRNA was also reduced. t-PA and PAI-1 were mainly expressed in syncytiotrophoblast membrane, and their immunostaining was increased in the pre-eclamptic placenta. The immunohistochemistry and western blotting analysis results showed that the expression of t-PA and PAI-1 increased in the PE group with PAI-1 higher than the t-PA. Subsequently, their mRNA expressions were increased in the pre-eclamptic placenta. Despite the increased t-PA levels in PE, fibrinolysis was suppressed, indicating the predominant role of reduced AnxA2 and overexpressed PAI-1 in suppressing PE's fibrinolytic mechanism. Previous studies have demonstrated that expression of t-PA, which is the target molecule of AnxA2, is actually increased in patients with pre-eclampsia^27^, indicating the predominant role of AnxA2 in t-PA activity.

Deficient AnxA2 dependent fibrinolytic pathway is correlated to increased intravascular thrombosis in both human disease and animal models. Several animal studies support the hypothesis that AnxA2 regulates hemostasis in-vivo. AnxA2 knockout mice, while displaying uncompromised development, fertility, and lifespan, accumulate fibrin in both intra and extravascular locations within the lungs, spleen, small intestine, liver, and kidney 28. AnxA2 alone, or combined with tPA, enhances vascular patency and reduces infarct size in several rodent strokes 29. Therefore, reduced AnxA2 and inactive tPA (action of which is reduced by decreased annexinA2 and very high levels of PAI-1) in the placenta of pre-eclampsia are responsible for increased fibrin deposition and reduced neo-vascularization. The evidence suggests that PAI-1 may play a role in cellular migration and invasion through the extracellular matrix on both normal processes like placentation and specific disease processes such as tumor invasion and metastasis. As we observed in our study, high levels of PAI-1 indicate poor prognosis from a variety of malignancies, including breast. Elevated levels of PAI-1 would result in fibrin deposition and occlusive lesions leading to thrombosis of the intervillous or spiral arteries and hence placental ischemia^30^. This is supported by the finding in the present study in which tPA and PAI-1 were significantly higher in the placenta of PE than the control placenta and the increased expression of PAI-1 than tPA in the pre-eclamptic placenta, suggests a decreased fibrinolytic mechanism in pre-eclampsia.

## Conclusion

Current study data illustrates that decreased AnxA2 with increased expression of tPA PAI-1, and altered association of AnxA2 with tPA in pre-eclamptic women's placental bed is culpable for altered fibrinolytic activity and plays a major role in the pathogenesis of pre-eclampsia. Given their well-established role in regulating angiogenesis and fibrin homeostasis, determining the fine details of cellular regulation of AnxA2, tPA and PAI-1 expression will likely contribute to a better understanding of normal placental biology and the pathogenesis of pre-eclampsia. However, there were some limitations in the study that, we could analyse the expression status of AnxA2, tPA, PAI-1 in the PE placenta compared to the normal but we have not assessed these proteins over the course of gestation to confirm the role of these proteins in the development of PE. The non-pregnant women were not investigated in this study. Hence, comparison of proteins between pregnant and non-pregnant females could not be done. Thus, the significance of the findings in normal pregnancy could not be verified.

## Figures and Tables

**Figure 3:**
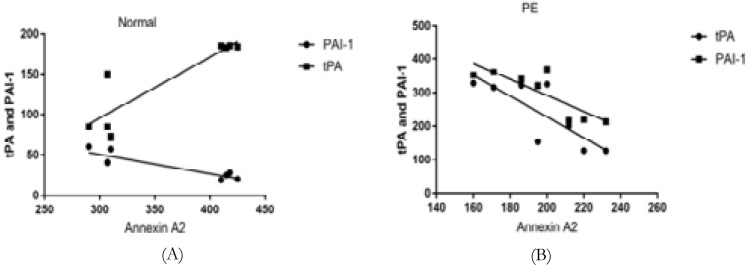
Correlation of AnxA2 with tPA and PAI-1 protein in Normal (A) and Pre-eclamptic placenta (B)

**Figure 4:**
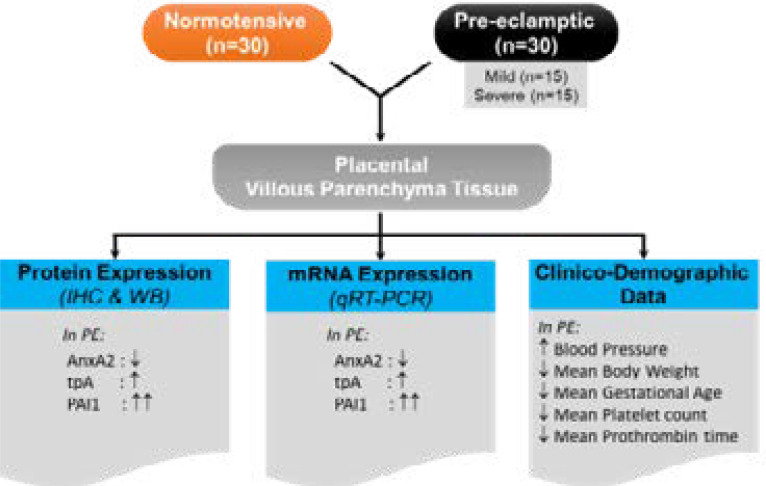
The diagrammatic representation of the study flow-chart.
